# *FMR1*, circadian genes and depression: suggestive associations or false discovery?

**DOI:** 10.1186/1740-3391-11-3

**Published:** 2013-03-23

**Authors:** Daniel F Kripke, Caroline M Nievergelt, Gregory J Tranah, Sarah S Murray, Katharine M Rex, Alexandra P Grizas, Elizabeth K Hahn, Heon-Jeong Lee, John R Kelsoe, Lawrence E Kline

**Affiliations:** 1 Department of Psychiatry, University of California, San Diego, La Jolla, CA 92093-0603, USA; 2 Scripps Clinic Viterbi Family Sleep Center, La Jolla, CA 92037, USA; 3 California Pacific Medical Center Research Institute, San Francisco, San Francisco, CA, 94107, USA; 4 Scripps Translational Science Institute, The Scripps Research Institute and Scripps Health, La Jolla, CA, USA; 5 Current address: Department of Pathology, University of California, San Diego, La Jolla, CA, 92093, USA; 6 CTICU, Children’s Hospital, Los Angeles, CA, 90029, USA; 7 Department of Psychiatry, Korea University College of Medicine, Seoul, South Korea; 8 Department of Psychiatry, VA San Diego Healthcare System, San Diego, CA, 92161, USA; 9 Institute of Genomic Medicine, University of California, San Diego, La Jolla, CA, 92093, USA

**Keywords:** Circadian, Depression, DSPS, *FMR1*, *PPARGC1B*, *GSK3B*, *NR1D1*, rs25702, rs28900, rs7732671

## Abstract

**Background:**

There are several indications that malfunctions of the circadian clock contribute to depression. To search for particular circadian gene polymorphisms associated with depression, diverse polymorphisms were genotyped in two samples covering a range of depressed volunteers and participants with normal mood.

**Methods:**

Depression mood self-ratings and DNA were collected independently from a sample of patients presenting to a sleep disorders center (1086 of European origin) and from a separate sample consisting of 399 participants claiming delayed sleep phase symptoms and 406 partly-matched controls. A custom Illumina Golden Gate array of 768 selected single nucleotide polymorphisms (SNPs) was assayed in both samples, supplemented by additional SNPlex and Taqman assays, including assay of 41 ancestry-associated markers (AIMs) to control stratification.

**Results:**

In the Sleep Clinic sample, these assays yielded Bonferroni-significant association with depressed mood in three linked SNPs of the gene *FMR1*: rs25702 (nominal P=1.77E-05), rs25714 (P=1.83E-05), and rs28900 (P=5.24E-05). This *FMR1* association was supported by 8 SNPs with nominal significance and a nominally-significant gene-wise set test. There was no association of depressed mood with *FMR1* in the delayed sleep phase case–control sample or in downloaded GWAS data from the GenRED 2 sample contrasting an early-onset recurrent depression sample with controls. No replication was located in other GWAS studies of depression. Our data did weakly replicate a previously-reported association of depression with *PPARGC1B* rs7732671 (P=0.0235). Suggestive associations not meeting strict criteria for multiple testing and replication were found with *GSK3B, NPAS2, RORA, PER3, CRY1, MTNR1A* and *NR1D1*. Notably, 16 SNPs nominally associated with depressed mood (14 in *GSK3B*) were also nominally associated with delayed sleep phase syndrome (P=3E10^-6^).

**Conclusions:**

Considering the inconsistencies between samples and the likelihood that the significant three *FMR1* SNPs might be linked to complex polymorphisms more functionally related to depression, large gene resequencing studies may be needed to clarify the import for depression of these circadian genes.

## Background

When the *PER* gene was shown to reside on the X chromosome in *Drosophila*, it was suggested that circadian period (tau) in humans might also be regulated by an X chromosome gene [1], explaining the higher prevalence of depression in women [2]. At this time, nothing was known of the circadian “clock genes” in humans, but a role for circadian phase and period disturbances in recurrent depression had been hypothesized [3,4], and evidence was increasing for a large genetic component in both unipolar and bipolar depression [5].

It now appears that there may be hundreds of mammalian genes which influence phase and timing of circadian rhythms [6]. Further, the clock-gene oscillator network influences transcription of what might be thousands of other clock-controlled genes which do not substantially feedback on the driving circadian oscillator [7]. Clock-controlled oscillation is influenced by transcription factors such as the CLOCK-ARNTL-CRY-PER complex binding at E-box sites, by NR1D1, NR1D2, and RORA and homologues interacting at RRE sites, by DBP and homologues binding with D-box sites, by kinases influencing clock component proteins, and by circadian control of energy metabolism [8], to give examples of an extremely complex network [9,10]. As a group, core clock genes and clock-controlled genes are significantly associated with bipolar disorder, with lithium response, and to some extent with unipolar depression, though the association of individual genes does not rise to conventional criteria for genome-wide association [11]. These significant associations appear to support the overall hypothesis that the circadian genetic system is involved in depression, implying an influence of combinations of numerous polymorphisms in complex genetic pathways.

Women have a lifetime prevalence of depression about twice that of men [2,12]. Among patients with delayed sleep phase, depression was strongly associated with delayed phase, and delayed phase has been reported in various samples of patients with depression and bipolar disorder [13-16]. Moreover, women with delayed sleep phase reported that their fathers were delayed significantly more often than did delayed sleep phase men, which might occur if a polymorphism causing delay could only be transmitted by fathers to female offspring because of its location on the X chromosome [13]. Of interest, knockouts of FMR1 on the non-autosomal portion of the X chromosome and its interacting paralog, FXR2, influence expression of behavioral circadian rhythms [17]. *FMR1* is well-known as the cause of fragile-X mental retardation, one of the most common inherited forms of intellectual disability [18]. Expansion of CGG repeats in the 5^′^ UTR of *FMR1* leads to reduced transcription of the FMRP protein. Mechanisms of FMR1 dysfunction at neural synapses, particularly glutamate synapses, have been extensively studied [18,19], but little is known about the circadian function of FMR1. Several reports describe excess lifetime depression among carriers of premutation fragile-X susceptibility [20-24]. Therefore, *FMR1* is a candidate for an X-linked circadian gene causing susceptibility to depression. With these rationales, *FMR1* SNPs were included in a circadian-gene screen of patients of the Viterbi Family Sleep Center. The same screen was also used on an independent sample of delayed sleep phase disorder. This report focuses on the association of circadian SNPs with depression symptoms, particularly *FMR1*.

## Methods

### Participants

From June, 2006 to May, 2010, 1281 patients undergoing polysomnographic evaluation at the Scripps Clinic Viterbi Family Sleep Center were recruited for a study of genetic factors in sleep disorders. All patients age 21 years or older whom staff could contact and who were willing and able to consent were included, along with rare younger adults of special interest. Written informed consent was obtained under the supervision of the Scripps Health IRB (Human Subjects Committee), but patients were not paid for research participation. Some results of this study have previously appeared elsewhere [25,26]. Participants’ mean age was 57.6 years (range 21 – 96) and 64.8% were male. As part of a long questionnaire gathering various symptoms relevant to sleep disorders, the QIDS-SR self-rating depression scale was obtained as an estimate of intensity of depression. The QIDS-SR has been shown to yield results highly correlated with the gold standard Hamilton Depression Rating Scale [27,28], but it is limited as a diagnostic scale. Structured determination of a lifetime history of major depression or bipolar disorder was not coded, though an unstructured psychiatric past history would be part of the usual clinical evaluation. DNA was purified from saliva collected in Oragene sampling kits (DNA Genotek Inc., Kanata, Ontario, Canada).

From May, 2004 to March, 2011, volunteers for a study of delayed sleep phase syndrome (DSPS) were recruited by newspaper, radio, word-of-mouth, and internet advertising in the San Diego region, and later by internet advertising throughout the United States. Control volunteers who had no delayed sleep phase or other prominent sleep disorder were likewise recruited, with targeting to match the affected participants by age, gender, and ethnicity. Both delayed sleep phase and control participants signed written informed consent for the collection and use of their DNA and were paid for their participation, under supervision of the UCSD Human Research Protections Program. The data of both studies were collected in accord with the principles of the Declaration of Helsinki. Participants also completed a series of questionnaires concerning their history of sleep disorders, especially delayed sleep phase symptoms, past medical and family history, psychiatric history, and also the QIDS-SR depression rating scale. More extensive descriptions of the participants have appeared elsewhere [13,26,29]. Participants contributed blood or saliva samples for DNA. Most of the samples were collected in Oragene saliva kits mailed to the participants. There were 460 participants recruited as delayed and 458 recruited as controls, but not all could be included because of phenotypic uncertainties, unsatisfactory DNA samples, genotyping quality control, and missing data. The N=805 sample usable for genetic association included 399 cases and 406 controls, 542 females (67.3%) and 263 males. Their mean age was 38 years (range 22 to 82).

### Genotyping

The major genotyping effort used a custom Illumina Golden Gate assay targeting 768 selected SNPs. Genotyping was performed following the manufacturer’s instructions. Genotypes were clustered within GenomeStudio using all samples with >95% call rates. SNPs with call rates <90%, or heterozygote frequencies >65% after re-clustering, were removed. Cluster positions for each SNP were manually inspected and edited if necessary. The final Illumina assay dataset from the sleep clinic contained 853 DNA samples genotyped in 630 SNPs (average call rate 99.7%). These were predominantly SNPs in circadian system genes targeted either for likely functional role or as linkage markers, with a few other SNPs included for suspected relevance to sleep disorders [30]. In addition, 41 SNPs selected as ancestry-informative markers (AIMs) were genotyped with Sequenom and SNPlex assays. Selected additional circadian SNPs were assayed with an additional custom SNPlex assay. DSPS-control samples were genotyped similarly, and a few polymorphisms including the *PER3* VNTR (rs57875989) were explored with supplemental Taqman assays. After screening for genotyping quality control, adequate heterozygosity, and Hardy-Weinberg equilibrium, there were 667 circadian-gene-related polymorphisms available for linear regression including 27 of the ancestry-informative markers. The ancestry-informative markers were used in combination with patient self-reports of ancestry to select those of European origin, when inclusion of those with nonEuropean ancestry produced an excessive genomic inflation factor. See Additional file 1 for SNP listings. In 6 DNA samples from our subjects, exons of the *FMR1* gene were kindly resequenced in Dr. S. T. Warren’s laboratory with the help of Dr. S. M. Bray, using the methods of Collins et al. [31].

DNA from 150 sib pairs (either both affected or one sibling with recurrent early-onset depression and one with no history of depression) were purchased from the NIMH Center for Collaborative Genetic Studies on Mental Disorders through the Rutgers University Cell and DNA Depository (GenRED 1, Study 7) [32]. Following PCR [33], the number of trinucleotide repeats in the FMR1 5^′^ UTR were estimated using an ABI 3130 sequencer to determine PCR fragment size.

### Association analysis

The associations of the sample polymorphisms with the QIDS-SR depression scale were determined using the linear regression module of PLINK [34]. Because the polymorphisms of most interest were located in the non-autosomal region of the X chromosome, males and females were analyzed independently. Then PLINK meta-analyses were used to combine results for the two genders and several studies. Significance was assessed with Bonferroni adjustments for testing multiple polymorphisms. For candidate-gene analyses, PLINK set-based tests were computed on linear regressions using default parameters of r^2^=0.5, P<0.05, and maximum number of SNPs=5, computing 10,000 permutations.

### Replication analysis

As replication samples, several published GWAS studies of SNPs associated with major depression were explored. The GenRED II data base was used to contrast patients with early-onset recurrent major depression to healthy controls selected according to Shi et al. from MDS samples [35]. Affected cases and controls were combined into a file encompassing 308 selected SNPs located in or near key circadian SNPs. The file of GenRED cases and MDS controls was processed with similar methods using PLINK linear regression and meta-analysis. Unfortunately, the circadian SNPs in our custom Illumina assays were poorly represented in the Affymetrix 6.0 arrays used for this GWAS, so there was little overlap. Association analyses of the GAIN Major Depression: Stage 1 Genome-wide Association In Population Based Samples Study (parent studies: Netherlands Study of Depression) [36-38] were downloaded from dbGAP and examined. In addition, Dr. Cathryn Lewis and Dr. Gerome Breen kindly searched previously computed associations of *FMR1* SNPs with 3 phenotypes of depression in three additional GWAS-phenotype studies, namely, case–control, [39] suicide attempt in depression cases, and quantitative trait for suicide [40]. Please see Additional file 2 for sources of DNA and genome-wide association data.

### Pleiotropy analyses

Participants in both the DSPS-control and Sleep Clinic samples were classified for the presence of probable delayed sleep phase syndrome, based on morningness-eveningness traits and a variety of clinical symptoms. Comorbidity with depression did not prevent delayed sleep phase classification unless depressive symptoms appeared first and appeared to cause the sleep disturbance. Details of the analyses of delayed sleep phase among study participants will be reported elsewhere, but here we consider pleiotropy of polymorphism-associated symptoms.

## Results

At the time participants were initially evaluated by the Sleep Clinic, QIDS-SR depression scores ranged from 0 to 25 (mean 7). A QIDS-SR score of 6 or more was reported by 46%, indicating at least mild depression, and 17% had a score of 11 or more, indicating at least moderate depression. Further, 1.7% (N=22) reported at least 5 symptoms of depression, meeting QIDS-SR criteria for current major depression. Whatever their depression status, almost all of these participants received at least one sleep disorders diagnosis, the great majority having at least a tentative diagnosis of some form of sleep apnea, upper airway resistance, or snoring. The next most common primary or comorbid diagnoses were insomnia of various forms, delayed sleep phase disorder, restless legs syndrome and/or period limb movements disorder, REM behavior disorders, and hypersomnia.

**Figure 1:**
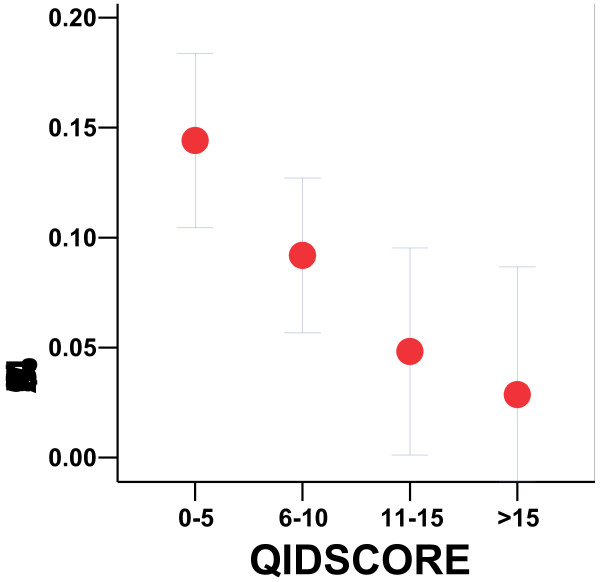
**Minor allele frequency versus QIDS score. **For the Sleep Clinic sample, the rs25702 minor allele frequency (MAF) with its 95% confidence interval range is plotted versus QIDS score ranges. QIDS scores correspond to the following levels of depression: mild (6–10), moderate (11–15), severe (16–20), and very severe (≥21) depression, where patients with scores <6 are not likely depressed and those >20 would usually not be observed in the clinic because they would need hospitalization. The lower the QIDS and the less depression observed, the more likely a patient was to have a minor allele.

**Figure 2:**
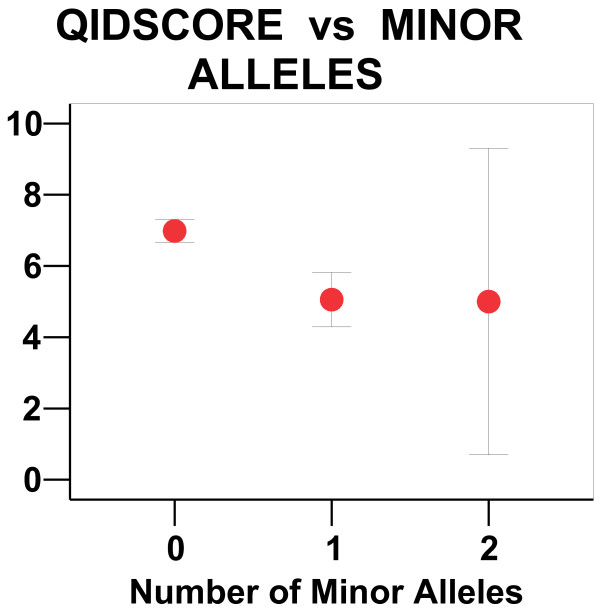
**QIDS scores for 0, 1, or 2 minor alleles. **For the Sleep Clinic sample, the QIDS depression score is plotted (with 95% confidence limits) for zero, one, or two minor alleles in rs25702. Since the sample was largely male, there were only 3 females (less than 1%) with two minor alleles in rs28900, rs25714, or rs25702. The small number with two minor alleles accounted for the large confidence limit in QIDS scores for that group. It appeared that women with one or two minor alleles had averaged about the same QIDS score, suggesting a dominant trait associated with lower QIDS score.

The linear regression analyses of Sleep Clinic participants of European origin (N=1086), combining separate analyses of each gender by meta-analysis, demonstrated significant association of QIDS-SR with 3 SNPs in *FMR1* after correction for multiple testing: rs25702 (nominal P=1.77E-05, Bonferroni P=.013 corrected for N=637 SNPs), rs25714 (P=1.83E-05, Bonferroni P=0.016), and rs28900 (P=5.24E-05, Bonferroni P=0.033). In males, minor allele frequencies were 0.083, 0.083, 0.058, respectively, and 0.075, 0.075, and 0.067 respectively in females. Odds ratios were -1.25, -1.25, and -1.36, respectively, the negative ratios indicating that the minor alleles were associated with absence of depression (Figures 1 and 2). The r^2^ values were 0.023, 0.023, and 0.030 respectively in females and respectively 0.027, 0.027, and 0.020 in males. These three SNPs were all in substantial linkage disequilibrium, which has been observed throughout the gene. The pairwise r^2^ linkage of rs25702 and rs25714 was 1.000; for rs 28900, the pairwise linkage with both rs25702 and rs25714 was r^2^=0.746. Eight of the 19 *FMR1* region SNPs analyzed displayed nominally-significant association with P<0.05, and all were to some extent linked. We did not identify haplotypes more significant than these single SNPs, and no significant interaction of *FMR1* SNPs with *FXR2* rs7211847 was observed. See Additional file 1 for further details.

**Figure 3:**
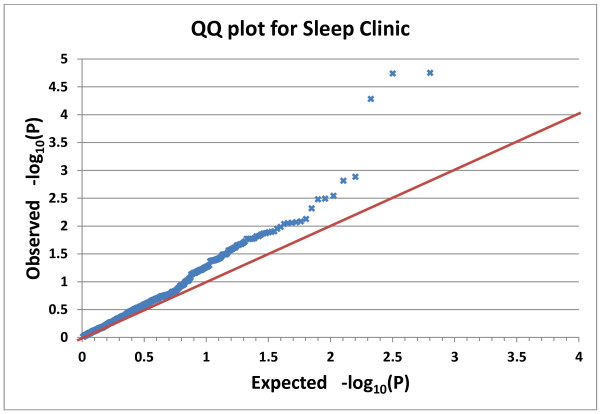
**Q-Q plot for Sleep Clinic SNPs. **The ranked observed and randomly-expected linear regression probabilities (P values) are plotted for the Sleep Clinic SNPs on a negative log_10 _scale. Higher values indicate smaller P values with greater significance. The three upper SNPs (from left to right) are *FMR1 *rs28900, rs25714, and rs25702. The red line indicates the expected trend were there no genomic inflation.

Of 634 SNPs in the Sleep Clinic analyses, 48 had nominal association with QIDS-SR at the P<0.05 level, but no SNPs in other genes met Bonferroni criteria (See Figure 3). Tests of association, with dichotomized depression phenotypes defined by QIDS-SR>10 or by meeting QIDS-SR criteria for current major depression, revealed similar trends with less impressive significance. The gene-wise set test for the association of *FMR1* with QIDS-SR was nominally significant with P=.0036, a probability which does not meet Bonferroni criteria for 36 genes tested (see Additional file 1, sheet 2).

In the DSPS cases and controls combined, linear regressions were performed on QIDS-SR as the dependent variable, covarying by age. Contrary to prediction, the genomic inflation factor was 1.0 for males and only 1.13 for females without selecting for European ancestry. Selection for participants of European ancestry or covarying by multidimensional scaling coefficients were not employed, because this did not further minimize genomic inflation. Association of polymorphisms with QIDS-SR using linear regression yielded 55 of 635 SNPs with nominal significance of P<0.05, but no SNPs overall approached Bonferroni significance and no *FMR1* SNPs had even nominal significance (all P>0.05). Analyses using dichotomized phenotypes defined by QIDS-SR>10 or lifetime history of depression (which could be estimated for this sample) yielded similarly non-significant associations when corrected for multiple testing. The gene-wise set test for the association of DSPS participants with QIDS-SR yielded nominal significance for *RORC*, *GSK3B*, and *CRY1*, none of which reached Bonferroni significance for 36 genes tested (Additional file 1, sheet 3). Note that the gene-wise set test results showed no consistency between the Sleep Clinic and DSPS samples.

Sequencing of coding exons of the *FMR1* gene in DNA from 6 of our subjects, courtesy of Dr. Warren’s laboratory, did not reveal any likely functional polymorphisms to which rs25714, rs25702, and rs28900 appeared in linkage disequilibrium.

In GenRED sib-pair DNA, the estimated number of *FMR1* 5^′^ UTR trinucleotide repeats averaged 24 in affected sibs and 24 in sibs without mental illness. There were no repeat lengths in the premutation range (>54 repeats) in either group.

Combining GenRED cases and MDS controls in a GWAS file, quality control yielded 722 cases and 710 controls among females and 298 cases and 910 controls among males. In our meta-analysis of GenRED GWAS males and females examined separately, only 4 SNPs were located in the *FMR1* region, including rs25714. For association with major depression, no FMR1 SNP was nominally significant, e.g., for rs25714, P=0.13, OR= -0.031. None of the 308 circadian SNPs examined approach Bonferroni significance for N=308 tested in males and females. With meta-analysis combining 6 linear regressions for males and females computed separately in each of the Sleep Clinic, DSPS, and GenRED-case-MDS-control samples, there were 52 SNPS in common (See Additional file 1, sheet 1). None of these 52 SNPs approached Bonferroni significance for N=52, and only 3 were nominally significant. In this meta-analysis of two genders in three studies, rs25714 in *FMR1* had no significant association with depression (P=0.066). However, when Sleep Clinic and DSPS data were combined for SNPs not analyzed in GenRed, several *FMR1* SNPs were significant, most notably rs25702 (P=0.00032). Indeed, rs25702 was the most nominally significant SNP in the entire meta-analysis, though falling short of Bonferroni significance.

In the GAIN depression study [36-38], data were available only for rs25714 among the *FMR1* SNPs that were highly significant in the Sleep Clinic study. For rs25714, the frequency of the minor allele was 0.059 in cases and 0.055 in controls (P=0.47, OR=1.086). In the depression and suicide GWAS studies of Lewis et al., 2010 [39] and Schosser et al., 2011, [40] FMR1 SNPs rs28900, rs25702, and rs25714 were not assayed by the Illumina 610 Quad bead chip, and there were no adequate proxies assayed.

In the pleiotropy analysis, of 614 SNPs for which meta-analysis results were available for association with both QIDS-SR and delayed sleep phase, there were 16 SNPs nominally significantly associated with both phenotypes (P=3E-06 by Fisher exact test of association, see Additional file 1, sheet 4).

## Discussion

To review, Bonferroni-significant associations of the QIDS-SR depression scale with 3 linked *FMR1* SNPs were found in the Sleep Clinic sample. Two tightly linked SNPs would meet Bonferroni criteria at the P<0.05 level for consideration of 637 SNPs, even adjusting for analyses of 3 separate studies. The reliability of these associations was supported by at least nominally significant association with 8 *FMR1* SNPs, by a nominally-significant gene-wise set test, and by nominally significant association in an *FMR1* paralog (*FXR2* rs7211847, P=0.0165 in Sleep Clinic and DSPS samples combined).

The functional plausibility of an *FMR1* association with depression is supported by its position on the non-autosomal X chromosome, by reports of fragile X premutation carriers being susceptible to depression [20-24], by well-known effects of FMR1 on brain synaptic functions [18,19], and by the interesting nominally-significant association in *FXR2* rs7211847, P=0.0165 in Sleep Clinic and DSPS samples meta-analysis) (see Additional file 1). Of the linked *FMR1* SNPs with most impressive association to depression, rs28900 may be part of a transcription factor binding site judging from ENCODE data, but no functional significance of rs25714 and rs25702 is apparent to us. Because the minor alleles of the 3 significant SNPs are associated with *lack* of depression, and because the minor allele frequencies are very different in Asians according to data from the 1000 Genome Browser [41], it seems plausible that these SNPs may not be functional but rather may be reciprocally linked to an unknown damaging polymorphism. Also, the significant associations noted were not replicated in the non-European portion of our samples. We were unsuccessful in identifying such a likely causal polymorphism linked to rs28900, rs25714, and rs25702 in the 6 DNAs we could sequence, but the sequencing did not encompass the promoter or some portions of the introns. Little is known about the effects of the expressed *FMR1-AS1* transcript (a RNA from the antisense strand of the *FMR1* promoter extending through the second exon) [42], various *FMR1* splice forms [18,43], or a copy-number insert involving much of the 3^′^ half of the gene and extending over 139,208 nucleotides 3^′^ (Variation_3272 in the Database of Genomic Variants) [44]. Thus, the 3 SNPs associated with QIDS-SR may be linked to larger *FMR1* variations of unknown complexity.

Significant association of *FMR1* SNPs was not replicated in the DSPS or GenRED samples. Further, meta-analysis of the 3 studies combined did not detect any significant *FMR1* polymorphisms, and the GAIN GWAS study likewise failed to locate nominally significant association with major depression in the *FMR1* region. Because the QIDS-SR is a measure of current-time depression, it is potentially insensitive to a prior history of depression, whereas the prior history is a crucial element of the lifetime depression phenotype usually preferred for GWAS studies. However, disparities between current and past depression phenotypes did not explain the failure to replicate using QIDS-SR in the DSPS-control sample, nor did exploring elements of the past history of depression collected for the DSPS sample result in stronger association. The DSPS and Sleep Clinic samples were recruited for differing comorbidities, with neither study focused primarily on depression. As a parametric phenotype, the QIDS-SR may have had certain advantages in statistical power over the case–control binary phenotypes used in the GWAS studies. Even combining many samples and exploring various depression phenotypes, GWAS studies of major depression have yielded few persuasive SNP candidates, generally implying either that depression may be associated with a large number of SNPs each accounting for little variance or that GWAS methods are somehow insensitive to “missing heritability” [45]. It is further possible that QIDS-SR scores may reflect qualitatively different aspects of depression as compared to research diagnoses of lifetime major depression, especially compared to recurrent depression with early onset (as in GenRED). Because the satisfactory evidence of statistical association for *FMR1* in the Sleep Clinic sample was not replicated in the 3 other studies of depression in which association could be examined, we must regard this as a suggestive association with import that can only be clarified with further study, recognizing that it may prove to be a false discovery.

The *FMR1* 5^′^ UTR triple repeat lengths in affected and unaffected GenRED sibs were approximately equal, apparently ruling out any frequent association of repeat expansion with early onset recurrent depression.

We have been unable to locate X chromosome data from several excellent GWAS studies of depression [46-49], so perhaps the X chromosome has not received sufficient attention. More attention to X would make sense in view of the evidence for sex-linking of depression [2,12]. Also, several polymorphisms in *MAOA* in the non-autosomal portion of the X chromosome appear associated with depression [50,51]. Although *ASMT* is found in the autosomal portion of the X and Y chromosomes, *ASMT* likewise has been little examined in GWAS studies despite expanding evidence associating *ASMT* with unipolar depression and bipolar disorder [26,52,53]. Both *MAOA* and *ASMT* may influence melatonin metabolism, with polymorphisms possibly leading to delayed melatonin offset, triggering photoperiodic mechanisms [54,55] which manifest as depression. Polymorphisms in *AANAT*, another component of melatonin metabolism participating in the circadian system, may also influence these mechanisms [56].

Remembering that none of the SNPs in the meta-analysis combining Sleep Clinic, DSPS, and GenRED participants (Additional file 1) reached significance after corrections for multiple testing, eight other circadian system genes besides *FMR1* were greatly over-represented among the nominally-significant results. There were 25 SNPs in *GSK3B* associated with depression with nominal P<0.05, falling into several linkage groups, and the gene-wise set test was nominally significant in the DSPS data set. This evidence supports previous reports that *GSK3B* has a role in affective responses to lithium and may be associated with bipolar disorder [57-62] and unipolar depression [63-65]. It may be relevant that *GSK3* interacts with fragile X syndrome [66]. There were 10 SNPs in *RXRA* with nominal significance in the DSPS sample, though only one of these was significant by meta-analysis with Sleep Clinic data. RXRA is a nuclear retinoic acid receptor with many interactions, e.g., it binds to ARNTL and CLOCK. There were 8 SNPs associated with depression with nominal significance in the *NPAS2* meta-analysis. NPAS2 is a functional homologue of CLOCK and likewise hybridizes with ARNTL. *NPAS2* has been associated with depression in previous reports, but the nominally significant SNPs in our report were not noted as suggestive in the previous work [46,67,68]. Also, there were 8 SNPs in *RORA* associated with depression with nominal P<0.05. *RORA* has been associated with depression in other reports, but we noticed no overlap among the SNPs reported of interest and the ones we recognized [67,69,70]. There were 3 SNPs in *PPARGC1B* significant in the meta-analysis including a previously-noted nominal association of rs7732671 with depression [71], herein replicated (P=0.024), but this SNP was not significant in the GAIN depression GWAS. Three SNPs in *CRY1* and *PER3* and 2 SNPs in the melatonin receptor *MTNR1A* were also nominally significant, but the well-known *PER3* VNTR (rs57875989) was not significantly associated with QIDS-SR in the DSPS sample, in which it was assayed. A *NR1D1* SNP that was previously found to be associated with bipolar disorder [71], rs2314339, was significantly associated with QIDS-SR by our meta-analysis, but we have not discovered any previous replication in unipolar depression studies. *FMR1, PPARGC1B, GSK3B, NPAS2, RORA, PER3, CRY1, MTNR1A* and *NR1D1* are circadian-related candidates for further study in relation to depression. Despite the unexpected number of nominally-significant SNPs in some of the genes mentioned, no persuasive haplotype associations in these genes were recognized with significance after correcting for multiple testing.

**Figure 4:**
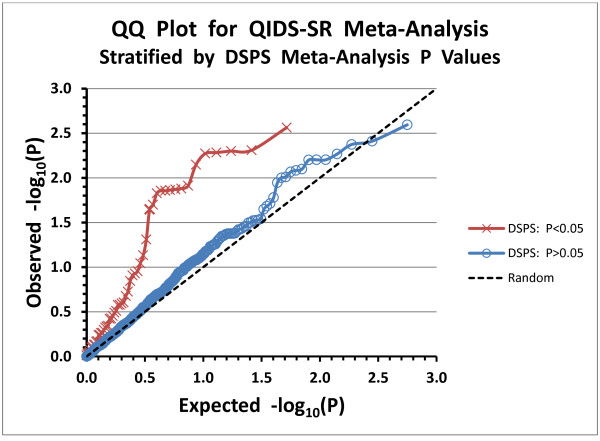
**Stratified Q-Q plot for SNPs Associated with QIDS-SR. **For SNP association with QIDS-SR, the ranked observed meta-analysis probabilities (P values) are plotted versus the expected probabilities. The scale is –log_10_(P), so that points above and to the right represent more significant (i.e. smaller) P values. Unlike Figure 3, only the 614 SNPs are plotted for which meta-analysis P values were available both for the QIDS-SR (Additional file 1, worksheet 1) and for DSPS classification in a dominant logistic genetic model. The P values for QIDS-SR association are stratified by whether the association of that SNP with DSPS was nominally significant (P<0.05). The expectation for 614 SNPs with random association is plotted in the black dashed line. Since the distance of the most deviant p values for P_DSPS_<0.05 (red line) is approximately 1 log unit from the random expectation (black dotted line) and from the line for P_DSPS_>0.05 (blue line), one may estimate that the probability of false discovery of pleiotropy for the most significant SNPs was ~0.10. *FMR1* SNPs were not included in Figure 4 because the dominant model could not be computed for this part of the X chromosome, but no *FMR1* SNP was significantly associated with DSPS in an additive model.

Andreassen et al. recently published very interesting methods for utilizing pleiotropy in SNP effects on comorbid phenotypes to leverage the power of association detection [72]. We have used their method of plotting stratified Q-Q data to illustrate evidence for pleiotropy of SNP effects (see Figure 4). Sixteen SNPs associated with delayed sleep phase had nominally significant pleiotropic effects on depression (Additional file 1, sheet 4). Comparing meta-analyses of QIDS-SR and DSPS association, using SNPs of the same participants in these two studies, we found comorbid association involving 14 *GSK3B* SNPs. *PPARGC1B* rs7732671 was also associated with both depression and delayed sleep phase, further supporting the functional role of this SNP. Finally, rs1800629, the well-known *TNF -*308 polymorphism, was associated with both phenotypes, supporting the previously-described influence of TNF upon depression and circadian rhythms [37,73]. From the ~1.0 log-unit divergence of the stratified Q-Q plots, it appears that individual SNPs did not meet false discovery criteria of P<0.05 for pleiotropy, but the graphic evidence and Fisher exact test for multiple-variant and polygenic pleiotropy were impressive. Studies of pleiotropic effects of circadian SNPs on circadian and depression phenotypes may have future application in discovering the causal polymorphisms influencing depression.

It is important to acknowledge some potential associations which were not confirmed. A preliminary result associating *TEF* rs738499 with depression [25] could not be confirmed in our expanded samples, despite an apparent replication in a group of Parkinson’s patients [74]. Our meta-analysis with Sleep Clinic data found no significant association of QIDS-SR with *OPN4* rs2675703 (P10L). Though the rs2675703 association in the DSPS data set was suggestive in meta-analysis by gender, it was not significant with combined genders either in additive or recessive models. The *CLOCK* C3111T SNP rs1801260 was not significantly associated with depression in these samples.

## Conclusions

In summary, these analyses point to several directions of interest without providing statistically rigorous and replicated evidence of SNPs associated with depression, apart from a modest replication of *PPARGC1B* rs7732671. When associations are found which are neither convincingly replicable nor persuasively insignificant, there will be no substitute for future study to determine if these are true or false discoveries. In the case of *FMR1*, because of the complex linkage of the SNPs studied so far, the antisense transcribed RNA, splice forms, and the copy number variation; we foresee no resolution of association issues without large-scale sequencing of the entire gene region in a larger sample of depressed patients.

## Abbreviations

ARNTL: Aryl hydrocarbon receptor nuclear translocator-like [HGNC:701]; ASMT: Acetylserotonin O-methyltransferase [HGNC:750]; CLOCK: Clock circadian regulator [HGNC:2082]; CRY1: Cryptochrome 1 (photolyase-like) [HGNC:2384]; D-box: DBP/E4BP4 binding element; DBP: D site of albumin promoter (albumin D-box) binding protein [HGNC:2697]; DSPS: Delayed sleep phase syndrome; ENCODE: Encyclopedia of DNA elements consortium data listed by UCSC genome bioinformatics; FMR1: Fragile X mental retardation 1 [HGNC:3775]; FMR1-AS1: *FMR1* antisense RNA 1 [HGNC:39081]; FMRP: The fragile X mental retardation (*FMR1*) protein; FXR2: Fragile X mental retardation, autosomal homolog 2 [HGNC:4024]; GAIN: Genetic association information network, denoting the GAIN major depression: stage 1 genome-wide association in population based samples study; GenRED: The NIMH genetics of recurrent early-onset depression project; GSK3B: Glycogen synthase kinase 3 beta [HGNC:4617]; GWAS: Genome-wide association study; HGNC: HUGO gene nomenclature committee at the European bioinformatics institute, http://www.genenames.org/; IRB: Institutional review board; MAOA: Monoamine oxidase A [HGNC:6833]; MDS: The NIMH molecular genetics of Schizophrenia project; MTNR1A: Melatonin receptor 1A [HGNC:7463]; NPAS2: Neuronal PAS domain protein 2 [HGNC:7895]; NR1D1: Nuclear receptor subfamily 1, group D, member 1 [HGNC:7962]; NR1D2: Nuclear receptor subfamily 1, group D, member 2 [HGNC:7963]; OPN4: Opsin 4 [HGNC:14449], previously melanopsin; OR: Odds ratio; PER: Period (*Drosophila melanogaster*) [GenBank:NC_004354]; PER3: Period circadian clock 3 [HGNC:8847]; PPARGC1B: Peroxisome proliferator-activated receptor gamma, coactivator 1 beta [HGNC:30022]; QIDS-SR: Quick inventory of depressive symptomatology self report; RORA: RAR-related orphan receptor A [HGNC:10258]; RORC: RAR-related orphan receptor C [HGNC:10260]; RRE: RevErbA/ROR binding element; RXRA: Retinoid X receptor, alpha [HGNC:10477]; SNP: Single nucleotide polymorphism; TEF: Thyrotrophic embryonic factor [HGNC:11722]; TNF: Tumor necrosis factor [HGNC:11892]; UTR: Untranslated region.

## Competing interests

The authors declare that they have no competing interests.

## Authors’ contributions

DFK designed the Sleep Clinic and DSPS data collection, helped select polymorphisms for assay, performed most statistical analyses, and wrote the manuscript first draft. CMN contributed to the design of the studies, selected polymorphisms for Illumina and AIMs assays, assisted assay interpretation and quality control, and participated in statistical analyses. GJT planned the Illumina Golden Gate assay design, selected genes and polymorphisms of interest, and arranged assay of DSPS samples in the UCSF Core laboratories. SSM oversaw the Golden Gate assay of the Sleep Clinic Samples and Sequenom assays of AIMs, called the Illumina genotypes for all participants, and participated in quality control and statistical design. KMR participated in DSPS study design, recruited and interviewed most of the DSPS study participants, built the DSPS clinical data base, and supported IRB oversight. APG and EKH recruited and interviewed most of the Sleep Clinic participants, built the Sleep Clinic study data base, and participated in study administration and IRB compliance. H-JL performed TaqMan assays and compiled much of the seasonal depression data base. JRK contributed to DSPS study design, supervised DNA purification as well as SNPlex and Taqman assays done in his laboratory, and consulted on preliminary results. LEK organized clinical research participation of Sleep Clinic physicians, interviewed and diagnosed patients, and arranged Scripps Clinic academic funding and staff support. All authors critiqued and approved the final manuscript.

## Supplementary Material

Additional file 1 **Summary of Sleep Clinic, DSPS, and GenRED data sets. **Worksheet 1, Meta-analyses and regressions: contains the main PLINK linear regression and meta-analysis outcomes for the polymorphisms analyzed. For the column key, see worksheet 1, cell B870. Worksheet 2, Sleep Clinic Set Test: shows the gene-wise PLINK set test results for the Sleep Clinic sample. Worksheet 3, DSPS Set Test: shows the gene-wise PLINK set test results for the DSPS study sample. Worksheet 4, Pleiotropy of Delayed Sleep Phase and Depression-Associated SNPs. Click here for file

Additional file 2 **Acknowledgement of GWAS sources. **Lists the sources, authors, and acknowledgements for GenRED, MDS controls used with GenRED, and GAIN GWAS data sets. Click here for file
