# Supplementary material for: FMR1, circadian genes and depression: suggestive associations or false discovery?
Source: J Circadian Rhythms. 2013 Mar 23;11:3. doi: 10.1186/1740-3391-11-3 (PMC3627611; doi:10.1186/1740-3391-11-3)
Supplement: Additional file 2 — Acknowledgement of GWAS sources. Lists the sources, authors, and acknowledgements for GenRED, MDS controls used with GenRED, and GAIN GWAS data sets. [file 1740-3391-11-3-S2.doc]

**Acknowledgement of GWAS sources**

**Study 7 (GenRED I)**

Data and biomaterials were collected in six projects that participated in the National Institute of Mental Health (NIMH) Genetics of Recurrent Early-Onset Depression (GenRED) project. From 1999-2003, the Principal Investigators and Co-Investigators were: New York State Psychiatric Institute, New York, NY, R01 MH060912, Myrna M. Weissman, Ph.D., and James K. Knowles, M.D., Ph.D.; University of Pittsburgh, Pittsburgh, PA, R01 MH060866, George S. Zubenko, M.D., Ph.D., and Wendy N. Zubenko, Ed.D., R.N., C.S.; Johns Hopkins University, Baltimore, MD, R01 MH059552, J. Raymond DePaulo, M.D., Melvin G. McInnis, M.D., and Dean MacKinnon, M.D.; University of Pennsylvania, Philadelphia, PA, RO1 MH61686, Douglas F. Levinson, M.D. (GenRED coordinator), Madeleine M. Gladis, Ph.D., Kathleen Murphy-Eberenz,

Ph.D. and Peter Holmans, Ph.D. (University of Wales College of Medicine); University of

Iowa, Iowa City, IW, R01 MH059542, Raymond R. Crowe, M.D. and William H. Coryell, M.D.; Rush University Medical Center, Chicago, IL, R01 MH059541-05, William A.Scheftner, M.D., Rush-Presbyterian.

**Study 52 (GenRED II)**

Data and biomaterials in this release were collected in six projects that participated in the National Institute of Mental Health (NIMH) Genetics of Recurrent Early-Onset Depression (GenRED) project (1999-2009). The Principal Investigators and Co-Investigators were: New York State Psychiatric Institute, New York, NY, R01 MH060912, Myrna Weissman, Ph.D.; Johns Hopkins University, Baltimore, R01 MH059552, J. Raymond DePaulo, M.D., and James B. Potash, M.D., M.P.H.; University of Pennsylvania, Philadelphia, PA (1999-2005), and Stanford University (2006-2009), R01 MH61686, Douglas F. Levinson, M.D. (GenRED coordinator); University of Iowa, Iowa City, IW, R01 MH059542e, Raymond R.Crowe, M.D., and William H. Coryell, M.D.; Rush University Medical Center, Chicago, IL, R01 MH059541-05, William A. Scheftner, M.D.; and University of Pittsburgh, Pittsburgh, PA (1999-2003), R01 MH060866, George S. Zubenko, M.D., Ph.D., and Wendy N. Zubenko, Ed.D., R.N., C.S.

**MDS controls**

Funding support for the companion studies, Genome-Wide Association Study of Schizophrenia (GAIN) and Molecular Genetics of Schizophrenia - nonGAIN Sample (MGS_nonGAIN), was provided by Genomics Research Branch at NIMH see below) and the genotyping and analysis of samples was provided through the Genetic Association Information Network (GAIN) and under the MGS U01s: MH79469 and MH79470. Assistance with data cleaning was provided by the National Center for Biotechnology Information. The MGS dataset(s) used for the analyses described in this manuscript were obtained from the database of Genotype and Phenotype (dbGaP) found at http://www.ncbi.nlm.nih.gov/gap through dbGaP accession numbers phs000021.v2.p1 (GAIN) and phs000167.v1.p1 (nonGAIN). Samples and associated phenotype data for the MGS GWAS study were collected under the following grants: NIMH Schizophrenia Genetics Initiative U01s: MH46276 (CR Cloninger), MH46289 (C Kaufmann), and MH46318 (MT Tsuang); and MGS Part 1 (MGS1) and Part 2 (MGS2) R01s: MH67257 (NG Buccola), MH59588 (BJ Mowry), MH59571 (PV Gejman), MH59565 (Robert Freedman), MH59587 (F Amin), MH60870 (WF Byerley), MH59566 (DW Black), MH59586 (JM Silverman), MH61675 (DF Levinson), and MH60879 (CR Cloninger). Further details of collection sites, individuals, and institutions may be found in data supplement Table 1 of Sanders et al. (2008; PMID: 18198266) and at the study dbGaP pages.

**Major Depression: Stage 1 Genomewide Association in Population-Based Samples**

Funding support for the GAIN Major Depression: Stage 1 Genome-wide Association In Population Based Samples Study (parent studies: Netherlands Study of Depression and Anxiety (NESDA) and the Netherlands Twin Register (NTR)) was provided by the Netherlands Scientific Organization (904-61-090, 904-61-193, 480-04-004, 400-05-717, NWO Genomics, SPI 56-464-1419) the Centre for Neurogenomics and Cognitive Research (CNCR-VU); the European Union (EU/WLRT-2001-01254), ZonMW (geestkracht program, 10-000-1002), NIMH (RO1 MH059160) and matching funds from participating institutes in NESDA and NTR, and the genotyping of samples was provided through the Genetic Association Information Network (GAIN). The dataset(s) used for the analyses described in this manuscript were obtained from the database of Genotypes and Phenotypes (dbGaP) found at http://www.ncbi.nlm.nih.gov/gap through dbGaP accession number phs000020.v2.p1. Samples and associated phenotype data for the GAIN Major Depression: Stage 1 Genome-wide Association In Population Based Samples Study (PI: Dr. Patrick F. Sullivan, MD, University of North Carolina) were provided by Dr. Dorret I. Boomsma, PhD and Dr. Eco de Geus, PhD VU University Amsterdam (PIs NTR), Dr. Brenda W. Penninx, PhD, VU University Medical Center Amsterdam, Dr. Frans Zitman, MD PhD, Leiden University Medical Center, Leiden, and Dr. Willem Nolen, MD PhD, University Medical Center Groningen (PIs and site-PIs NESDA).
